# Supplementary figures and images for: Epidemiology of malaria, schistosomiasis, and geohelminthiasis amongst children 3–15 years of age during the dry season in Northern Cameroon
Source: PLoS One. 2023 Jul 31;18(7):e0288560. doi: 10.1371/journal.pone.0288560 (PMC10389741; doi:10.1371/journal.pone.0288560)

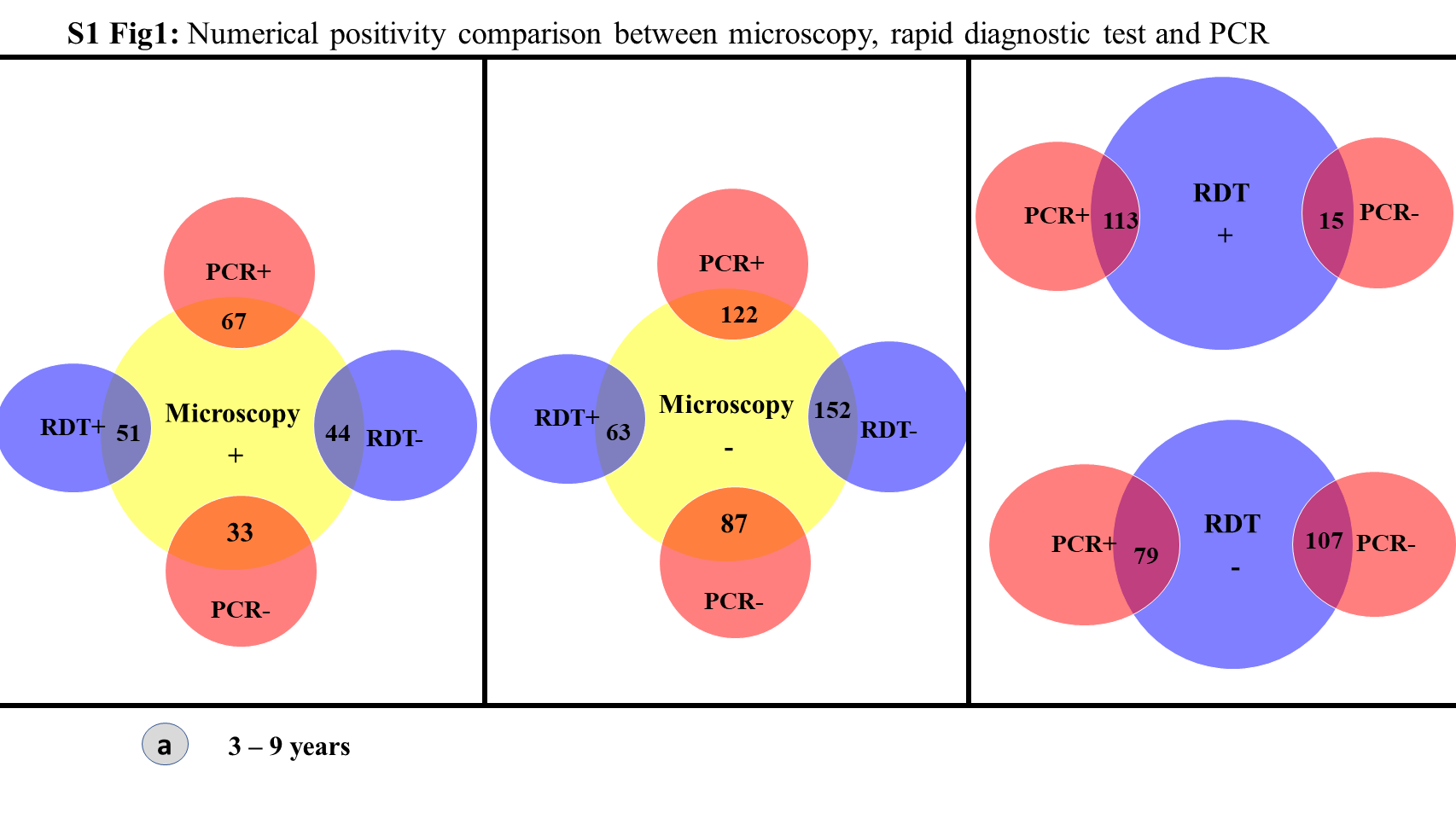

Supplement: S1 Fig — (TIF) [file pone.0288560.s001.tif]

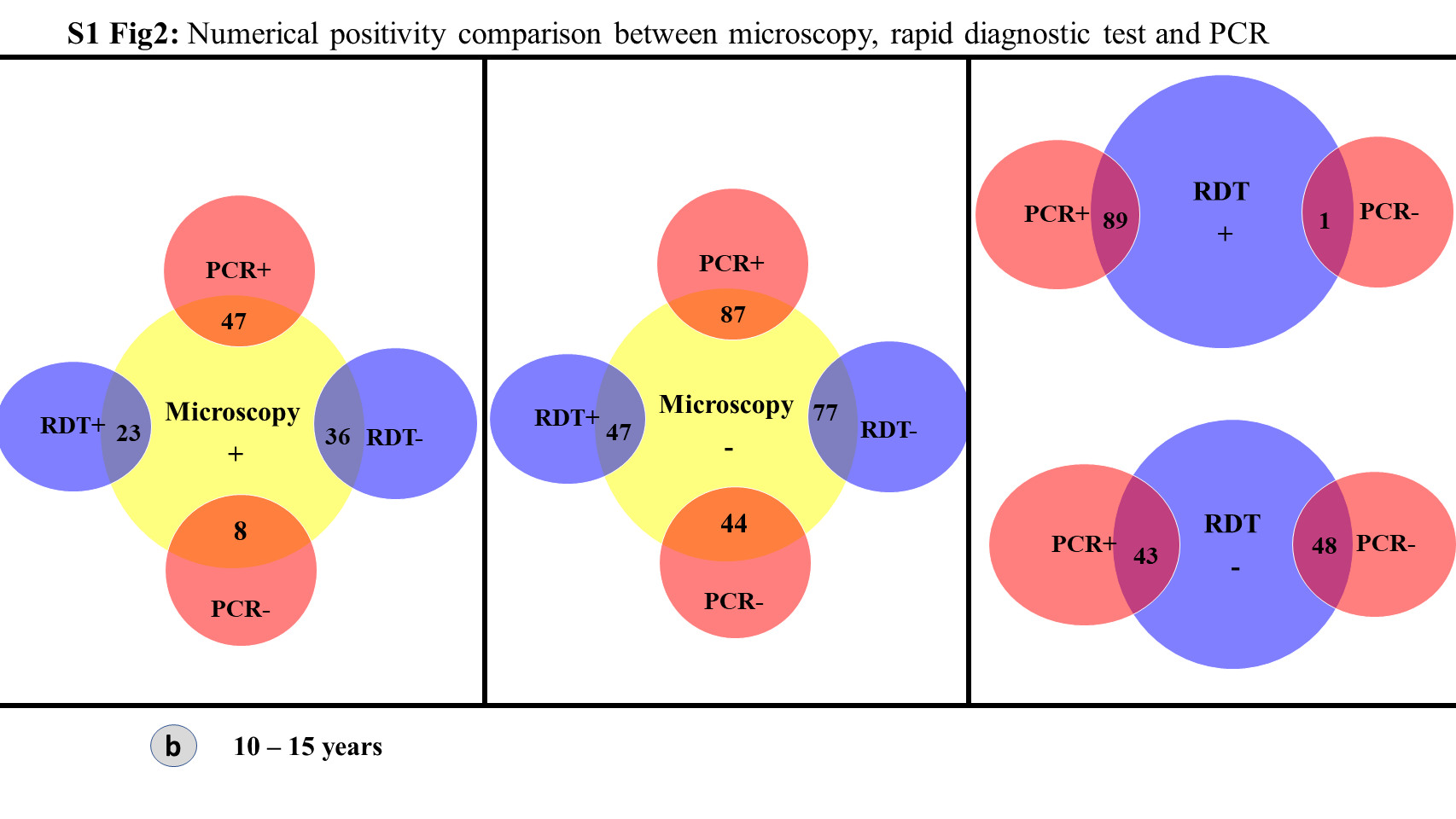

Supplement: S2 Fig — (TIF) [file pone.0288560.s002.tif]
