# Supplementary material for: Epidemiology of malaria, schistosomiasis, and geohelminthiasis amongst children 3–15 years of age during the dry season in Northern Cameroon
Source: PLoS One. 2023 Jul 31;18(7):e0288560. doi: 10.1371/journal.pone.0288560 (PMC10389741; doi:10.1371/journal.pone.0288560)
Supplement: S1 Table — (DOCX) [file pone.0288560.s003.docx]

| **Category** | | **Age (yr)** | | **Total** |
| --- | --- | --- | --- | --- |
| **Variables** | | **3-9** | **10-15** |  |
| % (n) | | 62.6 (310) | 37.4 (185) | 100 (495) |
| Schistosomiasis prevalence | | 75.8 (50) | 24.2 (16) | 13.3 (66) |
| Have you heard of bilharzia? | Yes  No | 20.7 (40)  89.4 (270) | 79.3 (153)  10.6 (32) | 39.0 (193)  61.0 (302) |
| Source of information about bilharzia | Radio/TV  School  CHW  Radio/School  Nil | 0.0 (0)  19.0 (32)  30.8 (04)  0.0 (0)  89.3 (274) | 100 (02)  81.0 (136)  69.2 (09)  100 (05)  10.7 (33) | 0.4 (02)  33.9 (168)  2.6 (13)  1.0 (05)  62.0 (307) |
| What causes bilharzia? | Sour food  Dirty water  River bath  Barefoot  Pig rearing  SF/DW  River/DW  SF/River  DW/Barefoot  SF/River/DW  Nil | 17.0 (08)  33.0 (32)  0.0 (0)  0.0 (0)  100 (02)  14.3 (03)  8.3 (01)  0.0 (0)  0.0 (0)  33.3 (01)  87.7 (265) | 83.0 (39)  67.0 (65)  100 (02)  0.0 (0)  0.0 (0)  85.7 (18)  91.7 (11)  100 (02)  100 (07)  66.7 (02)  12.3 (37) | 9.5 (47)  19.6 (97)  0.4 (02)  0.0 (0)  0.4 (02)  4.2 (21)  2.4 (12)  0.4 (02)  1.4 (07)  0.6 (03)  61.0 (302) |
| Community water source | Yes  No | 61.0 (230)  67.8 (80) | 39.0 (147)  32.2 (38) | 76.2 (377)  23.8 (118) |
| Type of water source for daily activities | Tap  Tap/Well  Well  Well/Stream  Tap/Stream | 51.3 (39)  69.0 (20)  54.4 (31)  71.5 (108)  61.5 (112) | 48.9 (37)  31.0 (09)  45.6 (26)  28.5 (43)  38.5 (70) | 15.4 (76)  5.9 (29)  11.5 (57)  30.5 (151)  36.8 (182) |
| Do you bathe in streams? | Yes  No | 63.3 (276)  57.6 (34) | 36.7 (160)  42.4 (25) | 88.1 (436)  11.9 (59) |
| Do you see blood in your urine? | Yes  No | 50.0 (12)  63.3 (298) | 50.0 (12)  36.7 (173) | 4.8 (24)  95.2 (471) |
| Feeling of blood in urine? | Normal  Depressed  Nil | 37.5 (03)  56.2 (09)  63.3 (298) | 62.5 (05)  43.8 (07)  36.7 (173) | 1.6 (08)  3.2 (16)  95.2 (471) |
| Do you feel pain upon urination? | Yes  No | 50.0 (11)  63.2 (299) | 50.0 (11)  36.8 (174) | 4.4 (22)  95.6 (473) |
| Abdominal pain? | Yes  No | 57.9 (11)  62.8 (299) | 42.1 (08)  37.2 (177) | 3.8 (19)  96.2 (476) |
| Itching urination (Females) | Yes  No | 74.2 (23)  62.5 (165) | 25.8 (08)  37.5 (99) | 6.3 (31)  53.3 (264) |
| Blood in feces? | Yes  No | 59.4 (19)  62.9 (291) | 40.6 (13)  37.1 (172) | 6.5 (32)  93.5 (463) |
| Urinate or excrete in streams? | Yes  No | 77.8 (140)  54.0 (170) | 22.2 (40)  46.0 (145) | 36.4 (180)  63.6 (315) |
| Presence of communal toilets? | Yes  No | 63.6 (164)  61.6 (146) | 36.4 (94)  38.4 (91) | 52.1 (258)  47.9 (237) |
| Presence of house toilet? | Yes  No | 61.1 (276)  79.1 (34) | 38.9 (176)  20.9 (09) | 91.3 (452)  8.7 (43) |
| Preferred site for excretion | Pit latrine  Bush  Stream  PL/Bush  Bush/Stream  PL/B/S | 54.1 (72)  71.2 (47)  64.7 (22)  60.8 (62)  100 (03)  68.8 (108) | 45.9 (61)  28.8 (19)  35.3 (12)  39.2 (40)  0.0 (0)  31.2 (49) | 26.9 (133)  13.3 (66)  6.9 (34)  20.6 (102)  0.6 (03)  31.7 (157) |
| Rearing domestic animal | Cow  Sheep  Goat  Cow/Goat  Cow/Sheep  Sheep/Goat  None | 34.3 (12)  57.5 (61)  47.3 (43)  25.0 (01)  75.0 (03)  39.2 (20)  80.9 (165) | 65.7 (23)  42.5 (45)  52.7 (48)  75.0 (03)  25.0 (01)  60.8 (31)  19.1 (39) | 7.1 (35)  21.4 (106)  18.4 (91)  0.8 (04)  0.8 (04)  10.3 (51)  41.2 (204) |
| Use of animal feces as fertilizers | Yes  No | 56.2 (77)  65.1 (233) | 43.8 (60)  34.9 (125) | 27.7 (137)  72.3 (358) |
| Walk/play barefoot? | Yes  No | 78.4 (276)  23.8 (34) | 21.6 (76)  76.2 (109) | 71.1 (352)  28.9 (143) |
| Handwashing with soap | Yes  No | 26.5 (48)  83.4 (262) | 73.5 (133)  16.6 (52) | 36.6 (181)  63.4 (314) |
| Do you often have diarrhea? | Yes  No | 97.8 (88)  54.8 (222) | 2.2 (02)  45.2 (183) | 18.2 (90)  81.8 (405) |
| How often do you receive treatment for worms? | 1X  Never | 52.8 (141)  74.1 (169) | 47.2 (126)  25.9 (59) | 53.9 (267)  46.1 (228) |
| Does your school have a health club? | Yes  No  No Response | 18.4 (07)  52.8 (159)  92.3 (144) | 81.6 (31)  47.2 (142)  7.7 (12) | 7.7 (38)  60.8 (301)  31.5 (156) |
| Opinion on health education clubs in primary school | Yes  No  No Response | 49.3 (166)  0.0 (0)  91.1 (144) | 50.7 (171)  0.0 (0)  8.9 (14) | 68.1 (337)  0.0 (0)  31.9 (158) |
| Opinion on health education using comic books | Yes  No  No Response | 49.3 (166)  0.0 (0)  91.1 (144) | 50.7 (171)  0.0 (0)  8.9 (14) | 68.1 (337)  0.0 (0)  31.9 (158) |
| Aware of periodic deworming? | Yes  No | 25.0 (30)  74.7 (280) | 75.0 (90)  25.3 (95) | 24.2 (120)  75.8 (375) |
| Parents administer anti-worm medicines | Yes  No | 51.5 (34)  64.3 (276) | 48.5 (32)  35.7 (153) | 13.3 (66)  86.7 (429) |
| Did you take PZQ last year? | Yes  No | 0.0 (0)  62.6 (310) | 0.0 (0)  37.4 (185) | 0.0 (0)  100 (495) |
| Intake of ABZ last year? | Yes  No | 0.0 (0)  62.6 (310) | 0.0 (0)  37.4 (185) | 0.0 (0)  100 (495) |
| Missed PZQ/ALB intake in school? | Yes  No | 65.1 (200)  58.5 (110) | 34.9 (107)  41.5 (78) | 62.0 (307)  38.0 (188) |

Key: CHW = Community Health Workers; SF = Sour Food; DW = Dirty Water; PL = Pit Latrine; B = Bush; S = Stream; PZQ = Praziquantel; ALB = Albendazole
