# Supplementary material for: Epidemiology of malaria, schistosomiasis, and geohelminthiasis amongst children 3–15 years of age during the dry season in Northern Cameroon
Source: PLoS One. 2023 Jul 31;18(7):e0288560. doi: 10.1371/journal.pone.0288560 (PMC10389741; doi:10.1371/journal.pone.0288560)
Supplement: S1 File — (DOCX) [file pone.0288560.s004.docx]

**Questionnaire**

Community name: ______________________ Community code: _______________

Participant name________________________________ ID_______/_______

**Section A: Sociodemographic data**

| Age (y) | Sex | Occupation | Class | T^0^ | Weight (Kg) | Height (m) | Years lived in village |
| --- | --- | --- | --- | --- | --- | --- | --- |
|  |  |  |  |  |  |  |  |

**Section B: Malaria**

| Have you ever heard of malaria? | Yes | No |
| --- | --- | --- |
| If Yes, where did you get the information about malaria?  Radio/TV School Health workers Community Never |  |  |
| History of fever for the past 03 days |  |  |
| Do you have a bed net in your house? | Yes | No |
| Do you sleep under a bed net? | Yes | No |
| How often do you sleep under a bed net?  Always Often Rarely Never |  |  |
| Have you taken any anti-malaria drug for the past 1 month? | Yes | No |
| What causes malaria? Mosquito bite Dirty water Witchcraft  Sour food Bathing in stream Rearing pigs Walking barefoot Don’t know |  |  |

**Section C: Schistosomiasis (or Bilharzia)**

| Have you ever heard of Bilharzia | Yes | No |
| --- | --- | --- |
| If Yes, where did you get the information about Bilharzia?  Radio/TV School Health workers Community Never |  |  |
| What causes Bilharzia (meaning of bilharzia in local language)  Witchcraft/Evil spirits Sour food Bathing in rivers Drinking dirty water Rearing pigs Mosquito bite Walking barefoot Don’t know |  |  |
| Is there any water source in your community? | Yes | No |
| What type of water source do you use for daily activities?  Tap Well Stream Rain |  |  |
| Do you bathe in stream/rivers? | Yes | No |
| Do you usually see blood in your urine? | Yes | No |
| How do you feel when u see blood in your urine?  Normal Sad Depressed |  |  |
| Do you feel pain (dysuria) when urinating? | Yes | No |
| Do you experience abdominal pain | Yes | No |
| Do you experience itching during urination (importantly for females) | Yes | No |
| Do you usually see blood in your feces? | Yes | No |
| Do you urinate or defecate in community streams | Yes | No |
| Are there communal toilets in your area? | Yes | No |
| Does your house have a toilet facility? | Yes | No |
| Preferred site you often excrete: Pit latrine Flush toilet Bush Stream |  |  |
| Do you people rear domestic animals such as: pig cow sheep goat | Yes | No |
| Do you people use animal feces to fertilize your farms? | Yes | No |
| Do you walk or play barefoot? | Yes | No |
| Do you eat raw food from the farm or eat unwashed foods/fruits? | Yes | No |
| Do you always wash your hands with or without soap after using the toilet? | Yes | No |
| Do you wash your hands with or without soap before eating? | Yes | No |
| Do you frequently have diarrhea? | Yes | No |
| Do you eat soils (usually brown soils on mud houses)? | Yes | No |
| How often do you receive treatment for worms?  Twice a year Once a year When I am sick Never |  |  |
| How many times do you bath a day?  Zero Once Twice It depends |  |  |
| Does your school has a health club? | Yes | No |
| Should primary schools have health education clubs on malaria and bilharzia? | Yes | No |
| Would comic cartoon books (picture-colouring books) on malaria & schistomosiasis educate you on these diseases? | Yes | No |

**Section D: Deworming history**

| Are you aware of the periodic deworming program? | Yes | No |
| --- | --- | --- |
| Does your parents usually give you worm medicine at home? | Yes | No |
| Did you take Praziquantel when your teacher was giving it last year? | Yes | No |
| Did you take worm medicine when you teacher was giving it last year? | Yes | No |
| Have you ever miss taking worm medicine when the teacher was giving it in school | Yes | No |
| Have your parents ever asked you not to take any medicine given to you in school | Yes | No |
