# Supplementary material for: Epidemiology of malaria, schistosomiasis, and geohelminthiasis amongst children 3–15 years of age during the dry season in Northern Cameroon
Source: PLoS One. 2023 Jul 31;18(7):e0288560. doi: 10.1371/journal.pone.0288560 (PMC10389741; doi:10.1371/journal.pone.0288560)

a **PCR gel image of *Plasmodium falciparum***

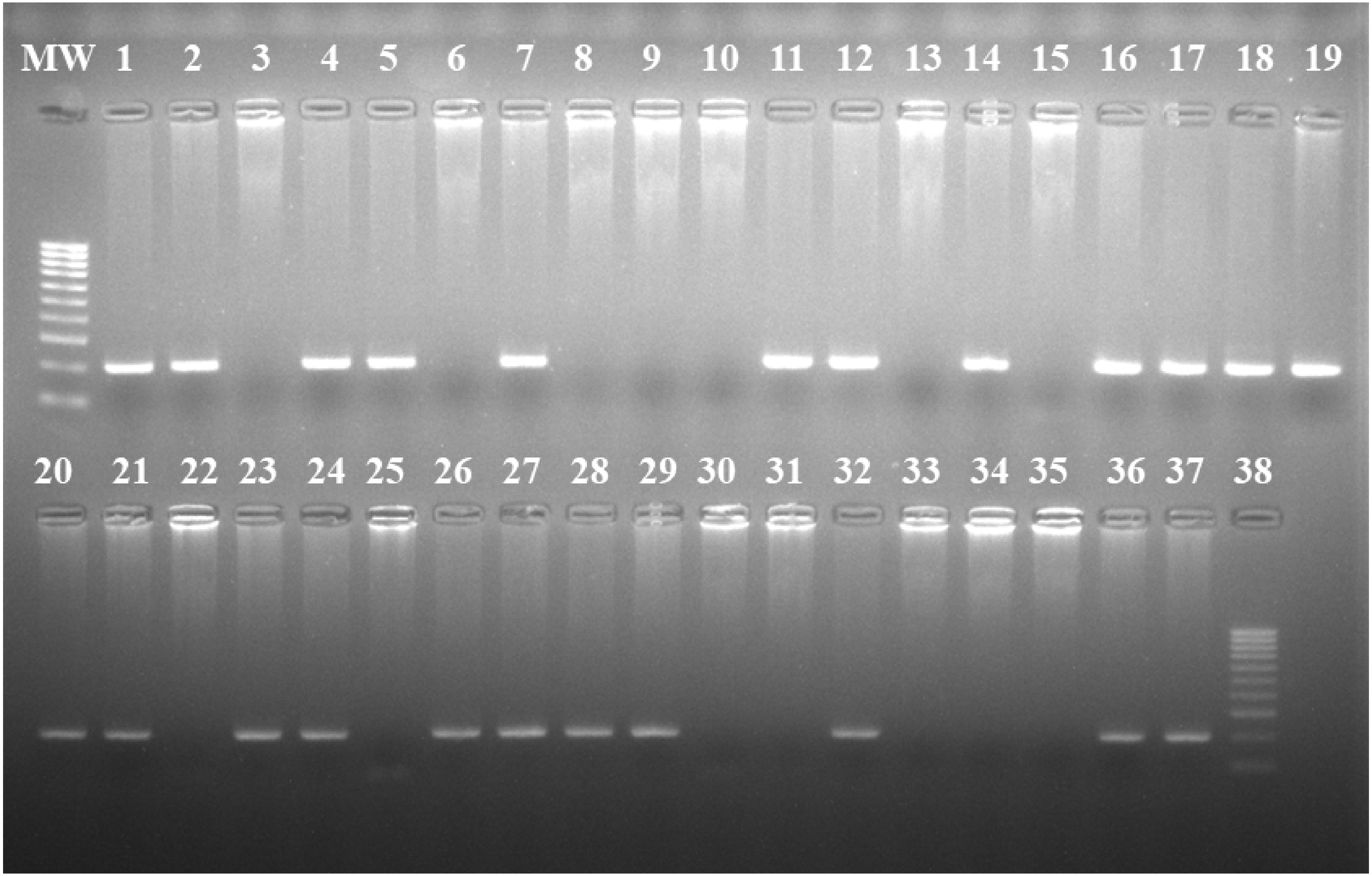

Where: MW = Molecular weight marker (100 bp)
Presence of bands = *P. falciparum* positive samples (205 bp)
No bands = *P. falciparum* negative samples

b PCR gel image of *Plasmodium malariae*

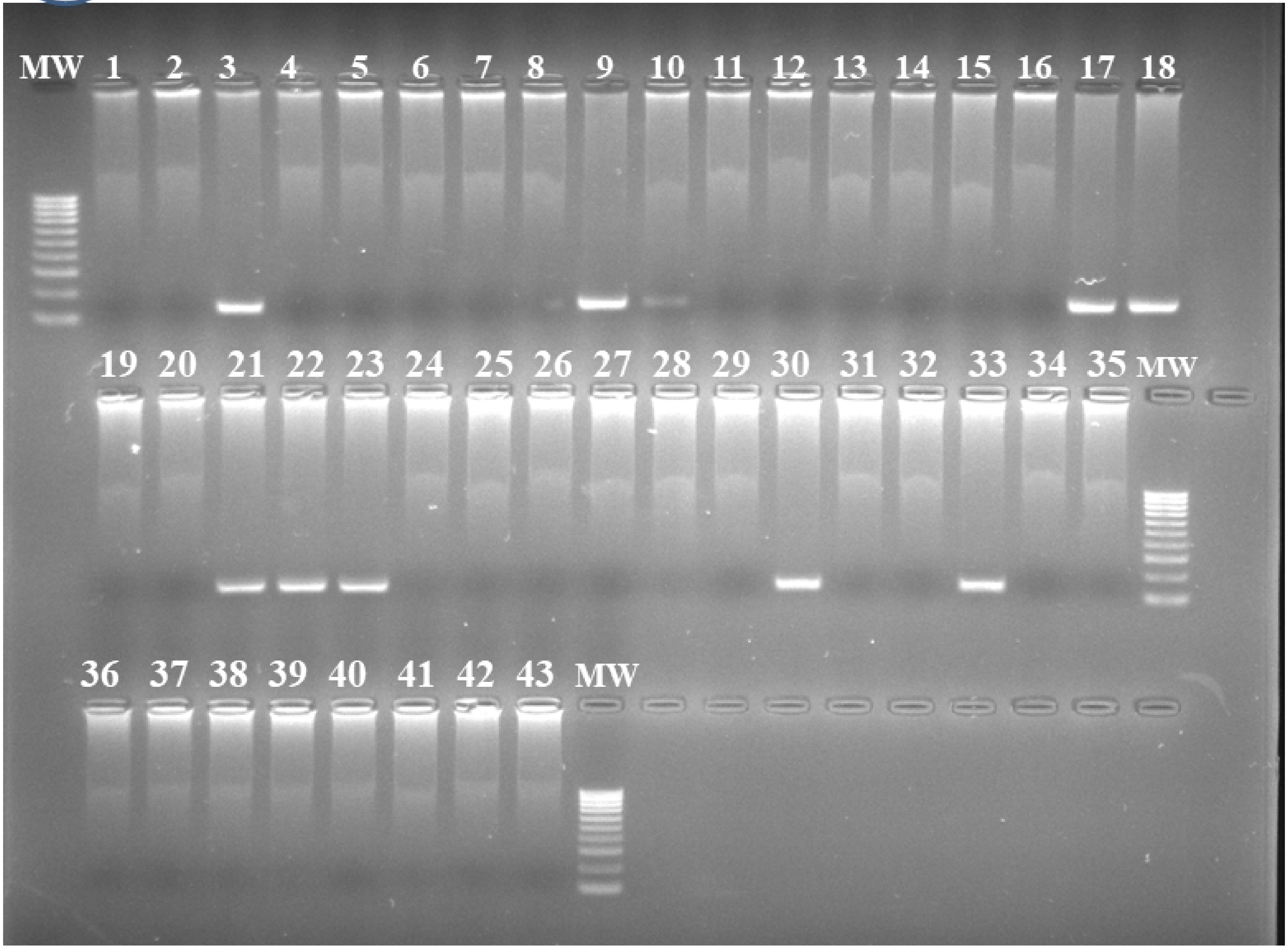

Where: MW = Molecular weight marker (100 bp)
Presence of bands = *P. malariae* positive samples (144 bp)
No bands = *P. malariae* negative samples

C *S. haematobium* eggs in urine

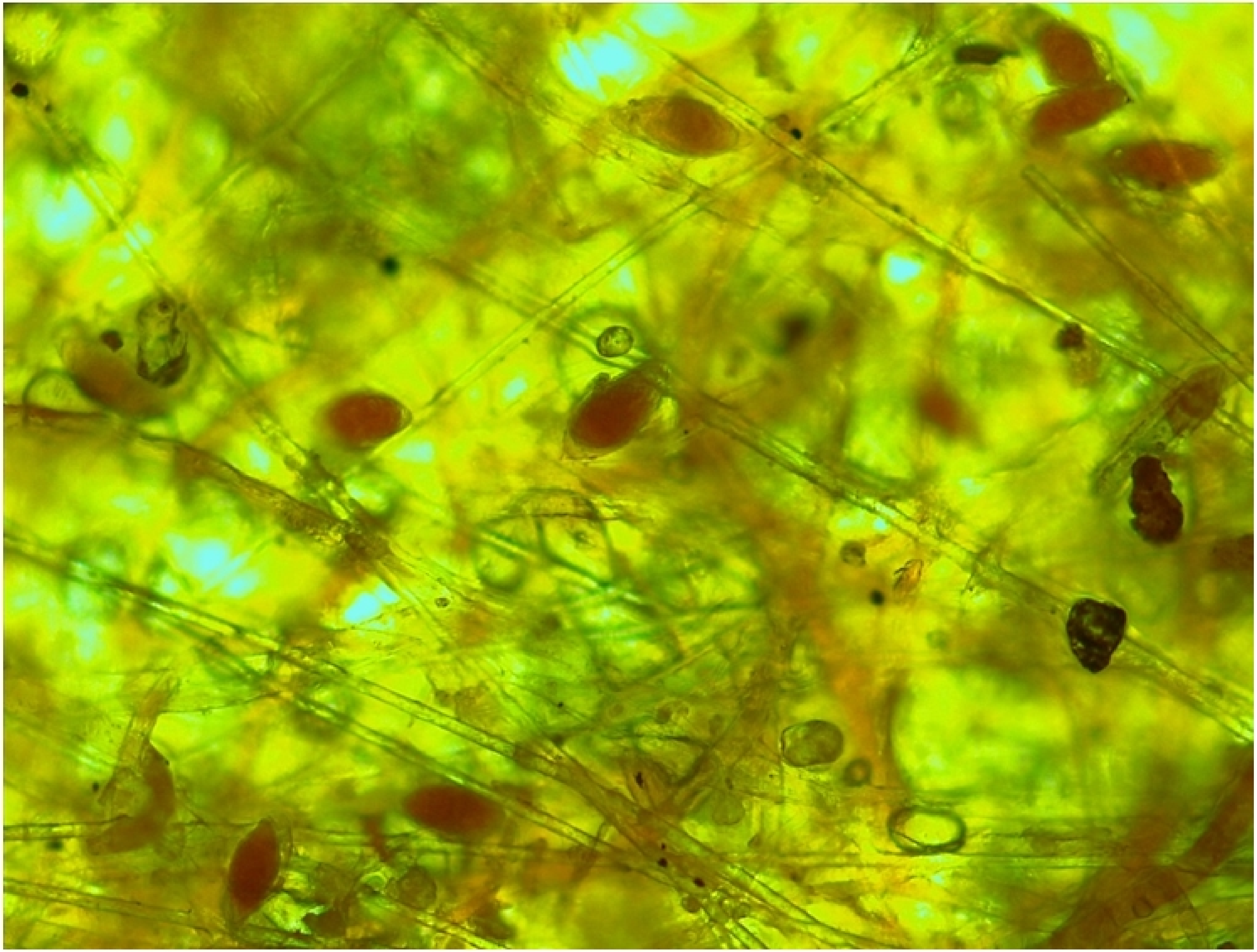

Supplement: S1 Raw images — (PDF) [file pone.0288560.s005.pdf]
