# Supplementary material for: Epidemiology of malaria, schistosomiasis, and geohelminthiasis amongst children 3–15 years of age during the dry season in Northern Cameroon
Source: PLoS One. 2023 Jul 31;18(7):e0288560. doi: 10.1371/journal.pone.0288560 (PMC10389741; doi:10.1371/journal.pone.0288560)
Supplement: Attachment — Submitted filename: Review comments.pdf [file pone.0288560.s006.pdf]

Persistent co-transmission of malaria, schistosomiasis, and geohelminthiasis among 3- 15 years old children during the dry season in Northern Cameroon

Review comments

The research is scientifically sound and addresses some of the challenges that programmes face during control of parasitic infections in the community. How to integrate control of malaria and helminthes will be interesting. At policy level it may be feasible.

Reading through some points can be addressed;

1. The introduction can be reduced to address only what is relevant to the study as it is.
2. Some of the methods should be clearly documented with slightly more details, ie, the urine filtration, Kato katz and the delivery of the questionnaire. All information is available, just package well.
3. Where a test did not give different results, or conflicting information such as the immunological then the authors can ignore (such results may be good for a thesis)
4. In the results, the tables are many, highlight the most important glaring difference and avoid many analytical information. The narrative should be brief and up to the point of difference.
5. The discussion can be reduced to be more specific to the research project and avoid repititition of what is obvious.
6. The references are many and probably, pick on the more recent.

Conclusion;

The manuscript can be polished and proceed with the publication.
