# Supplementary material for: Epidemiology of malaria, schistosomiasis, and geohelminthiasis amongst children 3–15 years of age during the dry season in Northern Cameroon
Source: PLoS One. 2023 Jul 31;18(7):e0288560. doi: 10.1371/journal.pone.0288560 (PMC10389741; doi:10.1371/journal.pone.0288560)
Supplement: Attachment — Submitted filename: Comments 11.05.23.docx [file pone.0288560.s008.docx]

1. “Health education talks on preventive measures for these diseases were delivered before each screening exercise. Furthermore, a questionnaire was administered to each participant.” Don’t you think that by giving them health education pertaining before any screening, it would have affected participants’ responses on the questionnaires? Or what did want to achieve by doing so?
2. I am so worried over the quality of responses from >5 years old children. Do 5 years old children able to comprehend the language in the questionnaire? Have they even ever heard of malaria, schistosomiasis or even remember their deworming history?
3. In definition of endpoints, have a sentence on symptomatic intestinal schistosomiasis as you did with urogenital schistosomiasis
4. Line 522, just change “frequent absence of consuming PZQ” to “poor PZQ uptake”
5. Revise comment 9: I have seen the S2 file. But an important piece of information is still missing in your methodology. Simply state the cut-off score of good KAP and bad KAP out of the total 37 variables. And a justification/basis of the decided cut-off

**Conclusion**: After all these have been addressed, the manuscript can proceed to publication
