# Supplementary material for: Epidemiology of malaria, schistosomiasis, and geohelminthiasis amongst children 3–15 years of age during the dry season in Northern Cameroon
Source: PLoS One. 2023 Jul 31;18(7):e0288560. doi: 10.1371/journal.pone.0288560 (PMC10389741; doi:10.1371/journal.pone.0288560)
Supplement: Attachment — Submitted filename: Rebuttal letter_Response to Reviewers comments_FNN.docx [file pone.0288560.s009.docx]

Date: 12 June 2023

**Response to Reviewers’ Comments**

1. “Health education talks on preventive measures for these diseases were delivered before each screening exercise. Furthermore, a questionnaire was administered to each participant.” Don’t you think that by giving them health education pertaining before any screening, it would have affected participants’ responses on the questionnaires? Or what did want to achieve by doing so?

**Response:** Health talks were delivered as part of explaining the purpose and objectives of the study. This is because participants immediately disperse to their daily activities once their samples have been collected and sometimes most do not even come back for the results or treatment (which is often given to the health worker). Therefore it was logical to deliver the health education lessons once the population assembled for recruitment. Indeed, the fact that the participants responded with poor KAP to malaria and schisto suggests that the health education talks did not impact the response to the questionnaire.

2. I am so worried over the quality of responses from >5 years old children. Do 5 years old children able to comprehend the language in the questionnaire? Have they even ever heard of malaria, schistosomiasis or even remember their deworming history?

**Response:** We previously answered this question in the first revision (line 131). Children < 5 years were intermittently assisted to respond to the question by their parents and this was made straightforward because the questionnaire sampling was led and verbally translated by indigenes who speak/understand both English and the local dialect (Fulfude). These children are aware of and have local appellations for these infections as it constitutes their daily health challenges. The deworming history of participants < 5 years was inferred from the parents (where necessary) and further confirmed by the class teachers.

3. In definition of endpoints, have a sentence on symptomatic intestinal schistosomiasis as you did with urogenital schistosomiasis

**Response:** Thank you for this remark. This has been included (line 304).

4. Line 522, just change “frequent absence of consuming PZQ” to “poor PZQ uptake”

**Response:** Correction done.

5. Revise comment 9: I have seen the S2 file. But an important piece of information is still missing in your methodology. Simply state the cut-off score of good KAP and bad KAP out of the total 37 variables. And a justification/basis of the decided cut-off

**Response:** As earlier mentioned (lines 199 – 202), for KAP variables, the cut-off score was between 0 – 1 with 0 = poor KAP and 1 = good KAP, particularly for the Yes/No questions. The basis of the decided cut-off was to categorize the data set and establish relevant epidemiological associations between the level of risk/exposure and outcome.

**Conclusion:** The authors appreciate the reviewer's comments to improve the scientific quality of the manuscript. We have adequately responded to the comments and hope the manuscript will now be considered for publication.
